# Pathological Characteristics of Pregnant Tree Shrews Infected by Zoonotic Hepatitis E Virus Genotype and the Effect of Estrogen on Virus Replication

**DOI:** 10.3390/vetsci12050483

**Published:** 2025-05-16

**Authors:** Peiying Zhu, Guojun Wang, Veerasak Punyapornwithaya, Chalita Jainonthee, Jijing Tian, Yan Liu, Fanan Suksawat, Sunpetch Angkititrakul, Yuchen Nan, Zailei Li, Xinhui Duan, Wengui Li

**Affiliations:** 1 College of Veterinary Medicine, Yunnan Agricultural University, Kunming 650201, China; peiying.z@kkumail.com (P.Z.); guojun.w@kkumail.com (G.W.); 18468111403@163.com (Y.L.); lizalei111@163.com (Z.L.); 2 Faculty of Veterinary Medicine, Khon Kaen University, Khon Kaen 40002, Thailand; sjirap@kku.ac.th (F.S.); sunpetch@kku.ac.th (S.A.); 3 Faculty of Veterinary Medicine, Chiang Mai University, Chiang Mai 50100, Thailand; veerasak.p@cmu.ac.th (V.P.); chalita.j@cmu.ac.th (C.J.); 4 College of Veterinary Medicine, China Agricultural University, Beijing 100193, China; jjt@cau.edu.cn; 5 College of Veterinary Medicine, Northwest A&F University, Yangling 712100, China; nanyuchen2015@nwsuaf.edu.cn

**Keywords:** hepatitis E virus, extrahepatic replication, estrogen, pregnant infection mode, adverse pregnancy, tree shrew

## Abstract

Hepatitis E, caused by the hepatitis E virus (HEV), is a zoonotic disease that extends beyond hepatocellular necrosis to replicate in multiple organs. While most infections are self-limiting, HEV infection during pregnancy is associated with severe outcomes, including acute liver failure, preterm delivery, and miscarriage, with the mechanisms underlying this high pathogenicity remaining poorly understood. This study established a pregnant tree shrew model with a late-stage HEV infection and a cellular model using zoonotic HEV genotypes GT3 and GT4 to investigate the effects of estrogen on HEV replication. Results showed that negative-strand RNA detection revealed replicative intermediates in feces and tissues during the acute phase, with peak viral loads occurring within one week and the highest titers in bile. Serum alanine aminotransferase (ALT) and aspartate aminotransferase (AST) levels rose at 3 days post-inoculation (DPI), peaking at 7 DPI. Elevated estrogen levels post-miscarriage correlated with increased viral loads, a trend mirrored in cell culture models showing linear relationships between estrogen and viral replication. Histopathology demonstrated viral hepatitis lesions in liver tissues and abnormalities in the uterus, ovaries, and brain, including hydropic degeneration, neuronal disruption, and granulosa cell necrosis. This study developed a pregnant tree shrew model for HEV infection, providing a robust tool for exploring pathogenic mechanisms during pregnancy and genotype-specific differences in zoonotic HEV pathogenicity. These findings offer new insights into the role of estrogen in HEV replication and its contribution to adverse pregnancy outcomes.

## Simple Summary

This study investigates how the Hepatitis E virus (HEV) affects pregnancy, with a particular focus on the role of estrogen in viral replication. Using a pregnant tree shrew model and cell cultures with HEV genotypes GT3 and GT4, we observed that viral loads peaked within a week, with the highest levels detected in bile. Liver enzyme levels (ALT and AST) increased early in the infection, and miscarriage was associated with elevated estrogen levels and enhanced viral replication. Furthermore, tissue analysis revealed liver damage and abnormalities in the uterus, ovaries, and brain. This model offers a valuable tool for studying HEV in pregnancy and underscores estrogen’s role in exacerbating infection.

## 1. Introduction

Hepatitis E (HE), caused by the hepatitis E virus (HEV), is an acute viral hepatitis that poses a significant threat to global human health [1]. Although most HEV infections are asymptomatic or self-limiting, symptomatic cases can involve jaundice, with severe outcomes observed in specific populations, particularly pregnant women. During late pregnancy, the mortality rate associated with an HEV infection can reach as high as 25–30% [2,3]. Annually, the mortality rate in pregnant women is significantly higher compared to non-pregnant women, at 19.8% and 1.9% [4]. An estimated 2400 to 3000 fetal deaths are attributed to HEV infection each year [5].

In healthy populations, an HEV infection is usually a self-limiting disease with a very low morbidity and mortality rate [5,6,7], while in some immunodeficient groups, such as organ transplant recipients and patients with HIV, an HEV infection may cause chronic hepatitis and cirrhosis of the liver [8,9]. Notably, a hepatitis E infection during pregnancy is a high-risk condition associated with severe maternal and fetal complications [10]. Maternal outcomes may include acute liver failure, preterm delivery, and maternal death, while fetal consequences range from malformations and intrauterine distress to growth restriction, preterm birth, and neonatal death due to jaundice, hepatosplenomegaly, respiratory distress, or sepsis [11,12]. Despite these serious implications, the mechanisms underlying the high pathogenicity of the HEV during pregnancy remain poorly understood.

Pregnant women exhibit increased susceptibility to an HEV infection, with viral loads significantly higher than in non-pregnant individuals. The elevated risks are hypothesized to result from a combination of factors, including viral genotype, hormonal fluctuations, and immune system adaptations during pregnancy [13,14,15,16]. However, progress in elucidating these mechanisms has been hindered by interspecies restrictions of HEV genotypes, challenges in viral isolation through cell culture, and the lack of robust experimental models.

HEV-3 and HEV-4 are zoonotic viruses, with pigs serving as the natural hosts for these two viral genotypes. Pigs infected with HEV generally do not exhibit obvious clinical symptoms, but they can carry the virus for extended periods and shed the virus through feces, saliva, etc., leading to contamination of the environment, water sources, or food (such as undercooked pork and pork liver). Humans may become infected with hepatitis E through consumption of contaminated food or close contact with these carrier pigs [17]. HEV-3 is spreading in industrialized countries such as Europe and the Americas, while HEV-4 is predominantly endemic in China [18]. The objective of this study was to demonstrate a late-stage pregnant tree shrew model and a complementary cellular infection model using zoonotic HEV genotypes GT3 and GT4. The model enables the investigation of estrogen’s role in HEV replication and its contribution to adverse pregnancy outcomes. The findings from this study provides an advanced understanding of the pathogenic mechanisms of HEV infection and support efforts to mitigate its burden, particularly in vulnerable populations.

## 2. Materials and Methods

### 2.1. Experimental Animals and Reagents

Eighteen healthy female tree shrews, aged 10–12 months and weighing 140–160 g, in late pregnancy, along with six healthy, non-pregnant female tree shrews, aged 10–12 months and weighing 110–130 g, were procured from the Laboratory Animal Center of the Kunming Institute of Zoology. Pregnancy confirmation and gestational staging were conducted by experienced animal care staff with over 10 years of expertise in breeding tree shrews. The diagnosis of pregnancy was based on post-mating behavioral changes (such as nesting and decreased mobility), progressive weight gain monitored after mating, and physical signs including abdominal distension and mammary gland enlargement. “Late pregnancy” was defined as approximately days 30 to 35 post-conception, representing the final 1–2 weeks of the typical 40–45-day gestational period in this species. During this phase, fetal development is near completion, and maternal physiological burden reaches its peak, making it a critical window to investigate virus–host interactions during pregnancy. Although methods such as ultrasonography or hormone assays would offer precise staging, they are currently not standardized for tree shrews; hence, expert phenotypic observation remains the accepted method in most existing studies involving this species. All animals were housed individually in separate cages to ensure controlled conditions. Ethical approval for all animal experiments was granted by the Science and Ethics Committee of Yunnan Agricultural University (Approval No. 202203079).

The reagents and kits used in this study, including reverse transcription kits and molecular quality standards, were obtained from Tiangen Biochemical Technology Beijing, China. In addition, 2× Trans Tag HIFI Mix II, SYBR Prime Ex Taq TII fluorescent dye and agarose were obtained from Sangon Bio tech, Shanghai, China. RNA extraction reagent TRIZOL and related molecular biology products were purchased from TransGen Biotech, Beijing, China. The biochemical kit for detecting aspartate aminotransferase (AST) and alanine aminotransferase (ALT) levels was purchased from Nanjing Jiancheng Bioengineering Research Institute, the estrogen detection kit was purchased from Mingzhi Biotechnology Shanghai, China., and the hematoxylin and eosin (H&E) staining kit was obtained from Servicebio Technology, Wuhan, China.

For experimental infections, a fecal sample with a high titer of HEV genotype 4 was used to infect the tree shrews. Transinfection studies in the A549 cell line were conducted using a type 3 HEV clone (Kernow-C1).

### 2.2. Procedure for HEV Infection in Tree Shrews

A 10% suspension of HEV-positive swine feces was prepared in phosphate-buffered saline (PBS), supplemented with 1% penicillin–streptomycin to prevent bacterial contamination. The mixture was stored at 4 °C overnight, centrifuged at 6000 rpm for 20 min at 4 °C, and the resulting supernatant was filtered through a 0.22 μm membrane. The filtered supernatant was quantified for initial viral copy number by real-time PCR and stored at −80 °C for future use.

A total of 24 tree shrews were randomly divided into two groups: the challenge group (12 late-pregnancy females) and the control group (6 late-pregnancy females and 6 non-pregnant females). All animals were housed individually in isolated cages to maintain hygienic conditions and prevent cross-contamination. The challenge group received 500 μL of the prepared virus suspension via intraperitoneal injection, while the control group was administered an equivalent volume of sterile normal saline. A second dose of the virus or saline was given at 2 days post-inoculation. The animals’ diet and health status were closely monitored daily throughout the experiment.

To evaluate the progression of the infection, a total of four tree shrews were euthanized at each time point (3, 7, 14, 21, 28, and 35 days post-inoculation, DPI), including two from the challenge group and two from the control group (one pregnant and one non-pregnant in each group). Tissue samples collected for molecular analysis were stored at −80 °C, while samples for histopathological examination were fixed in 4% paraformaldehyde. Blood samples were collected and centrifuged at 3000 rpm for 10 min to separate serum for subsequent analyses.

### 2.3. Liver Function Analysis

Liver function was assessed using commercial kits for AST and ALT, following the manufacturer’s protocols. A standard curve was generated to determine enzyme levels. Optical density (OD) was measured at 510 nm for each sample, and AST and ALT concentrations were calculated based on the standard curve.

### 2.4. Estrogen Level Measurement

Estrogen levels were quantified using an enzyme-linked immunosorbent assay (ELISA) kit in accordance with the manufacturer’s instructions. A calibration curve was established, and the optical density (OD) of each well was measured at 450 nm to determine estrogen concentrations in the samples.

### 2.5. Histopathological Examination

Tissue samples fixed in 4% paraformaldehyde were processed for histopathological analysis. The tissues were dehydrated, embedded in paraffin, and sectioned into 4 μm thick slices. Sections were stained with HE for microscopic evaluation. All slides were examined using an Olympus BH-2 microscope to identify histopathological changes.

### 2.6. Effect of Estrogen on Kernow-C1 Replication in A549 Cells

To simulate estrogen levels during different pregnancy stages, various concentrations of estrogen analogs—diethylstilbestrol (DES), 250 pg/mL, 3500 pg/mL, and 11,000 pg/mL—were added to A549 cell cultures [19]. Cell viability was evaluated using a CCK-8 assay. After three generations of blind passage, the Kernow-C1 HEV strain was treated with repeated freeze–thaw cycles. After being filtered through a 0.22 μm membrane, the initial concentration of the virus solution was determined using real-time PCR; then, the suspension was inoculated into the A549 cell monolayer with 100 μL per well. Following a 2 h incubation at 37 °C with periodic agitation, the supernatant was removed, and cells were washed with PBS. Maintenance medium containing varying concentrations of DES was then added. At 3 DPI, cells were harvested, and the supernatant underwent three additional freeze–thaw cycles before storage at −80 °C for further analysis.

### 2.7. Quantification of HEV Titer

The HEV titer was quantified using primers designed based on gene sequences retrieved from GenBank, synthesized by Sangon Bioengineering Technology, 5418F [forward, 5′-GGTGGTTTCTGGGGTGAC-3] and 5418R [reverse, 5′-GARAAGAGAGTGYCGCTGGA-3′] [20]. Amplification was performed using these HEV-specific primers. The resulting PCR products were separated on a 1% agarose gel, and the 70 bp target band was excised and purified. This purified product was ligated into the pMDTM19-T vector and transformed into DH5α cells for Original TA Cloning Kit (TA Cloning). Positive clones were identified via colony PCR, and plasmid DNA was extracted using a commercial plasmid extraction kit, following the manufacturer’s instructions.

Serial tenfold dilutions of the HEV-positive plasmid, ranging from 10^−1^ to 10^−8^, were prepared to generate a standard curve for HEV quantification by real-time PCR. The HEV copy number in tissue samples was determined using the standard curve and the corresponding cycle threshold (Ct) values obtained from real-time PCR analysis.

For each organ sample, a fixed tissue mass of 50 mg was used for total RNA extraction with TRIzol reagent according to the manufacturer’s instructions. The RNA was eluted in 50 µL of RNase-free water, and 1 µL of this eluate was used for reverse transcription in a 20 µL reaction. Real-time PCR was then performed using 1 µL of the resulting cDNA per reaction. Viral loads were expressed as “copies/µL”, where µL refers to the cDNA volume used in the qPCR reaction. Although this method maintains consistency across tissue samples by standardizing input mass and reaction volumes, we acknowledge that expressing viral loads in “copies/µL” may not fully account for differences in RNA yield or tissue-specific extraction efficiency. For future comparative studies between tissues, normalization to tissue mass or total RNA concentration (e.g., copies/mg or copies/µg RNA) may offer a more accurate representation of viral distribution.

At each time point, tissues from two infected tree shrews were collected (*n* = 2 biological replicates). RNA was extracted from each tissue sample, and real-time PCR was performed in technical duplicates. The average of the technical replicates was used as the representative value for each biological sample. The error bars in viral quantification figures represent the standard deviation across biological replicates.

To verify viral replication, the presence of negative-sense RNA, a replication intermediate, was assessed using nested PCR. For the detection of negative-sense RNA as evidence of replication, nested PCR was performed using primers that generated a 348 bp product, external primer set 3156N [forward, 5′-AATTATGCC(T)CAGTAC(T)CGG(A)GTTG-3′] and 3157N [reverse, 5′-CCCTTA(G)TCC(T)TGCTGA(C)GCATTCTC-3′] and internal primer set 3158N [forward, 5′-GTT(A)ATGCTT(C)TGCATA(T)CATGGCT-3′] and 3159N [reverse, 5′-AGCCGACGAAATCAATTCTGTC-3′] [21].

## 3. Results

### 3.1. Clinical Observations and Pathological Changes

**Figure 1:**
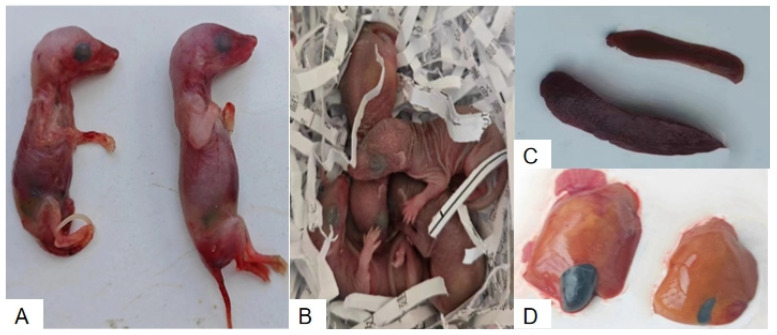
Comparison of fetal outcomes and organ pathological changes between HEV-infected and control pregnant tree shrews. (**A**) Stillborn fetuses from the HEV-infected group. (**B**) Healthy fetuses from the control group. (**C**) Enlarged spleens from the HEV-infected group compared to the control group. (**D**) Enlarged and necrotic livers from the HEV-infected group compared to normal livers in the control group.

Clinical observations revealed no difference for the number of fetuses between experimental and control group. However, at 5 DPI, stillbirths were observed exclusively in the experimental group. Some stillborn fetuses displayed stiffness and significant reductions in body weight, as shown in Figure 1A. The stillbirth rate in the experimental group was 33%. In contrast, healthy fetuses with normal morphology were delivered in the control group (Figure 1B). No HEV RNA was detected in the tissues of stillborn fetuses by RT-qPCR, suggesting an absence of vertical transmission in this model. In the experimental group, incomplete detachment of fetal membranes was noted in some cases, accompanied by maternal hemorrhage, resulting in a maternal mortality rate of 17%. Postmortem examinations revealed pathological changes, including necrosis and enlargement of the liver and spleen, as well as abnormal distension of the gallbladder (Figure 1C,D). In contrast, no abnormalities were detected in the control group.

### 3.2. Quantification of HEV Replication in Tissues

**Figure 2:**
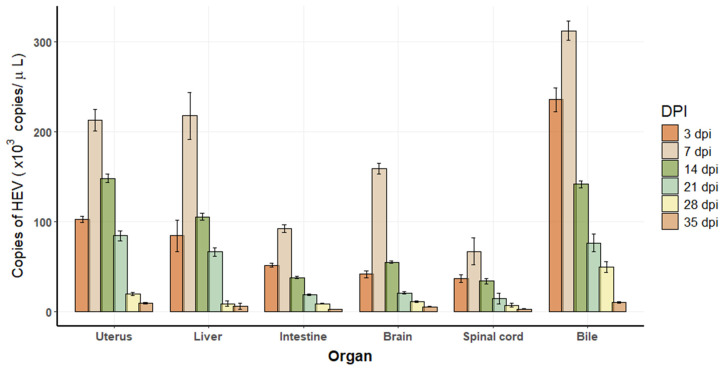
Viral replication in tissues of HEV-infected tree shrews. Each time point represents *n* = 2 biological replicates. Each biological sample was tested in technical duplicate, and the average was used for quantification. Error bars indicate standard deviations of biological replicates.

A standard curve generated for HEV detection via real-time PCR demonstrated an inverse relationship between cycle threshold (Ct) values and HEV copy numbers. The regression equation, y = −3.4615x + 37.816, exhibited a high correlation coefficient (R^2^ = 0.9993) and amplification efficiency of 96.55%. The real-time PCR results showed that the initial concentration of the viral solution was 1.24 × 10^2^ copies/uL. From 3 DPI, a 348 bp negative-strand RNA fragment was confirmed to be detected in the feces and various tissues of the infected tree shrew, indicating a viral replication intermediate. Viral loads were quantified in various organs, including the uterus, liver, intestine, brain, spinal cord, and bile, at different DPI: 3, 7, 14, 21, 28, and 35 (Figure 2). The highest viral titer was detected in bile, followed by the uterus and liver, while the lowest viral loads were observed in the spinal cord.

### 3.3. Serum ALT and AST Dynamics in Liver Function Tests

**Figure 3:**
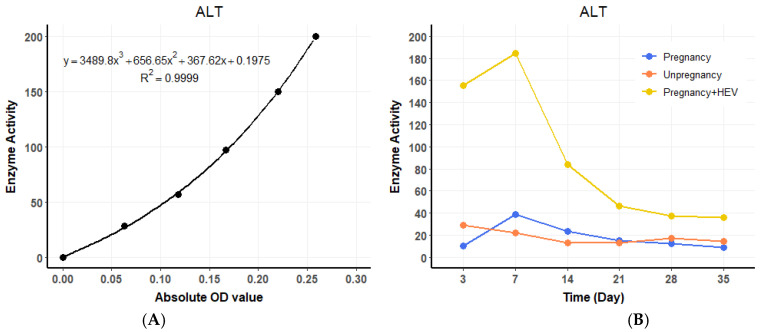
ALT dynamics and standard curve. (**A**) Standard curve for ALT enzyme activity based on OD values. (**B**) Temporal changes in serum ALT levels across groups over the experimental period. For the HEV-infected group, values represent the average of two biological replicates (*n* = 2) per time point. For the control groups (pregnant and non-pregnant), one animal (*n* = 1) per time point was used.

**Figure 4:**
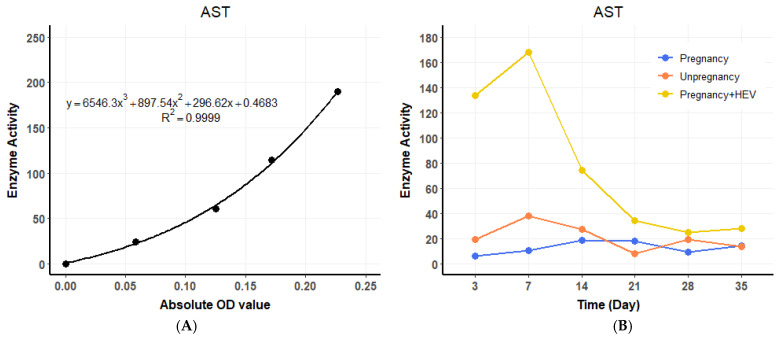
AST dynamics and standard curve. (**A**) Standard curve for AST enzyme activity based on OD values. (**B**) Temporal changes in serum AST levels across groups. For the HEV-infected group, values represent the average of two biological replicates (*n* = 2) per time point. Control groups were based on one sample (*n* = 1) per time point.

ALT levels in the HEV-infected pregnant group began to rise at 3 DPI, showing a 4.4 to 8.4 times increase compared to the pregnant and non-pregnant control groups, respectively. A standard curve for ALT enzyme activity was established using OD values (Figure 3A), and temporal changes in ALT levels were plotted across experimental groups (Figure 3B). ALT levels peaked at 7 DPI before gradually declining to baseline levels by the end of the experimental period. Similarly, AST levels exhibited a consistent increase, starting at 3 DPI in the HEV-infected pregnant group, with values 16 to 8.4 times higher than those observed in the pregnant and non-pregnant controls, respectively. A standard curve for AST activity was generated (Figure 4A), and dynamic changes in AST levels were illustrated over time (Figure 4B). AST levels also reached their maximum at 7 DPI before progressively returning to baseline. The trends in ALT and AST levels reflect the impact of HEV infection on liver function during pregnancy.

### 3.4. Dynamic of Serum Estrogen

**Figure 5:**
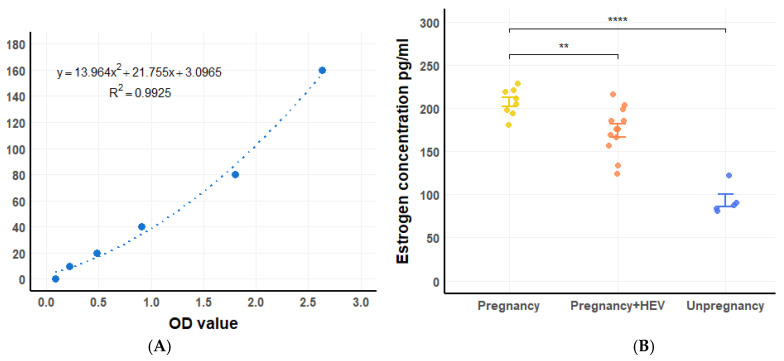
Estrogen levels and standard curve. (**A**) Standard curve for estrogen determination using OD values. (**B**) Estrogen concentrations in pregnant, HEV-infected pregnant and non-pregnant tree shrews. Significant differences are denoted (** *p* < 0.01, **** *p* < 0.0001).

Estrogen levels were measured using an ELISA-based approach, with a standard curve established to calculate hormone concentrations (Figure 5A). During pregnancy, estrogen levels rose significantly, with notable differences observed between HEV-infected and control pregnant tree shrews (*p* < 0.01) and between pregnant and non-pregnant groups (*p* < 0.0001) (Figure 5B). In the HEV-infected group, two tree shrews experienced abortion at 3 DPI and died at 5 DPI. Interestingly, estrogen levels increased following abortion, contrary to expectations of a decrease, and this rise coincided with increased viral loads.

### 3.5. Histopathological Changes in HEV-Infected Pregnant Tree Shrews

**Figure 6:**
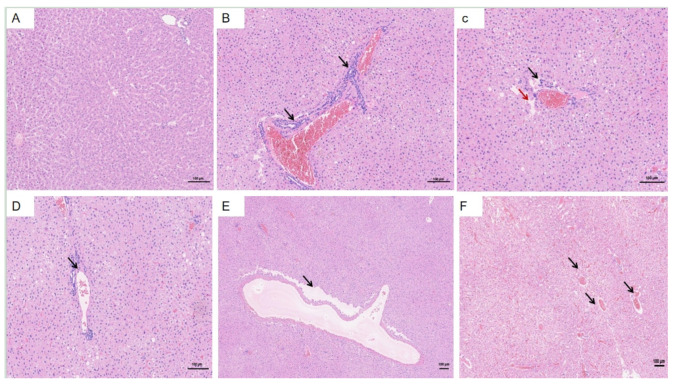
Histopathological changes in the liver of HEV-infected pregnant tree shrews. (**A**) Normal hepatic architecture observed in the control group. (**B**–**D**) Lymphocytic infiltration around blood vessels in the HEV-infected group. (**E**) Disappeared hepatocytes. (**F**) The central hepatic sinusoids of the liver lobules are highly dilated and stagnant. The scales of different line segments in the figure represent 100 μm.

The liver was selected as the primary organ for observing pathological changes associated with HEV infection. In the HEV-infected group, typical viral hepatitis lesions were identified, which were absent in the control group (Figure 6A). Key pathological features observed in the infected group included disorganized hepatic cords and lymphocytic infiltration around blood vessels (Figure 6B–D). Loss of hepatocytes around the central vein (Figure 6C, red arrow, Figure 6E). Additionally, Kupffer’s cells are increased, the central hepatic sinusoids of the hepatic lobules are highly dilated and stagnant, and the hepatocytes are atrophied or have even disappeared (Figure 6F). These findings indicate significant hepatic damage caused by HEV genotype 4 infection, further highlighting the liver’s susceptibility to viral replication and associated immune responses during pregnancy.

**Figure 7:**
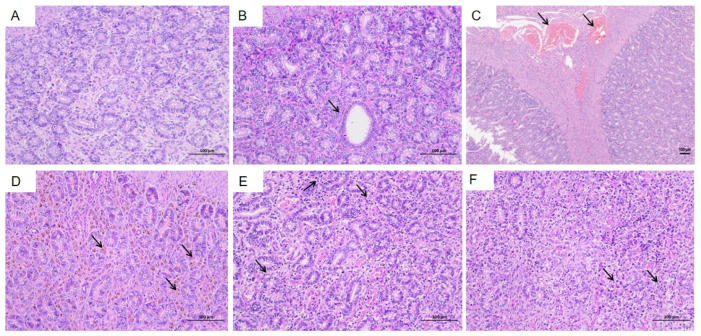
Histopathological changes in the uterus of HEV-infected tree shrews. (**A**) Normal uterine structure observed in the control group. (**B**) Dilation of uterine glands in the HEV-infected group. (**C**) Bleeding from the fundus of the uterus. (**D**) Brown pigment deposition in the interstitial tissue. (**E**,**F**) Irregular arrangement of uterine glands and scattered infiltration of granulocytes and lymphocytes in the stromal tissue. The scales of different line segments in the figure represent 100 μm.

The uterus and ovaries were examined due to their potential involvement in abortion observed in the HEV-infected group (Figure 7A). Infected tree shrews exhibited notable pathological changes in the uterus compared to the control group. Key findings included dilation of uterine glands (Figure 7B, black arrow), Heavy bleeding from the uterine fundus (Figure 7C, black arrows), accompanied by hydropic degeneration in a small portion of glandular epithelium. Affected cells displayed swelling, loose cytoplasm with light staining, and structural irregularities. Brown pigment deposition was observed in the interstitial tissue (Figure 7D, black arrows). Additionally, irregular arrangements of uterine glands were evident, along with scattered infiltration of granulocytes and lymphocytes within the stromal tissue (Figure 7E,F, black arrows). These histopathological changes highlight the impact of HEV infection on reproductive organs and its association with adverse pregnancy outcomes, such as abortion.

**Figure 8:**
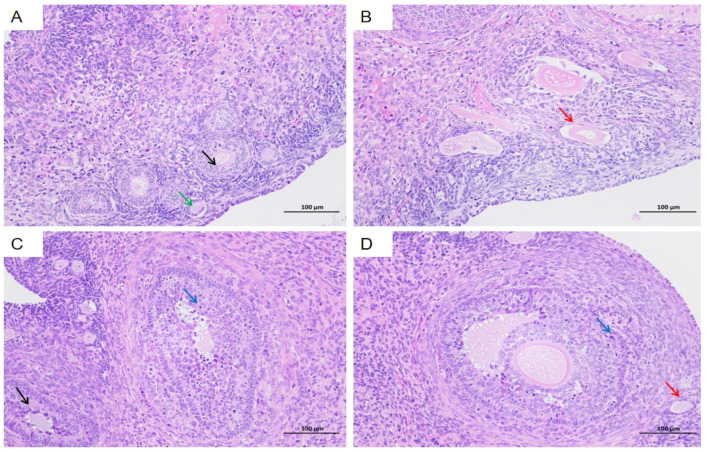
Histopathological changes in the ovaries of HEV-infected tree shrews. Primary follicles (**A** green arrow), secondary follicles (**A** and **C** black arrow), and atretic follicles (**B** and **D** red arrow) are visible. Granulosa cell necrosis (**D** blue arrow) is evident in some follicles, with wrinkled and deeply stained nuclei. The scales of different line segments in the figure represent 100 μm.

Histological examination of the ovaries revealed the presence of primary follicles (Figure 8A, green arrow), secondary follicles (Figure 8A, black arrow), and atretic follicles (Figure 8B,D, red arrow). Occasional necrosis of granulosa cells was observed within the follicles (Figure 8C,D, blue arrow), characterized by wrinkled and deeply stained nuclei. Notably, no significant inflammatory cell infiltration was detected in the ovarian tissues.

**Figure 9:**
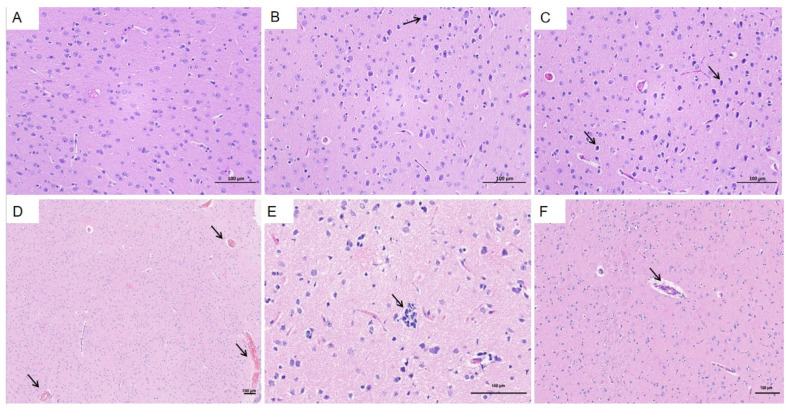
Histopathological changes in the brain of HEV-infected tree shrews. (**A**) Normal brain tissue in the control group. (**B**,**C**) Dilation and congestion of meningeal blood vessels and cortical vascular cuffs in the HEV-infected group. (**D**–**F**) Irregularly arranged neurons with deep staining, shrunken nuclei, and poorly defined nuclear–cytoplasmic boundaries in the HEV-infected group. The scales of different line segments in the figure represent 100 μm.

The brain was identified as a primary extrahepatic site of HEV replication. In the HEV-infected group, notable pathological changes were observed compared to the control group (Figure 9A). These included dilation and congestion of meningeal blood vessels, along with softening of the molecular layer adjacent to the meninges. Extensive lymphocytic infiltration was evident, accompanied by microglial proliferation. Additionally, multiple vascular cuffs were observed in the cortical region, characterized by congested blood vessels (Figure 9B,C, black arrows). The arrangement of neurons in the brain tissue was irregular, with some neurons displaying deep staining, shrunken nuclei, irregular shapes, and poorly defined boundaries between the nuclei and cytoplasm (Figure 9D,F, black arrows). These histopathological findings highlight the significant impact of HEV infection on brain tissue, potentially contributing to extrahepatic complications.

### 3.6. The Effect of Estrogen on HEV Replication in Cell Culture

**Figure 10:**
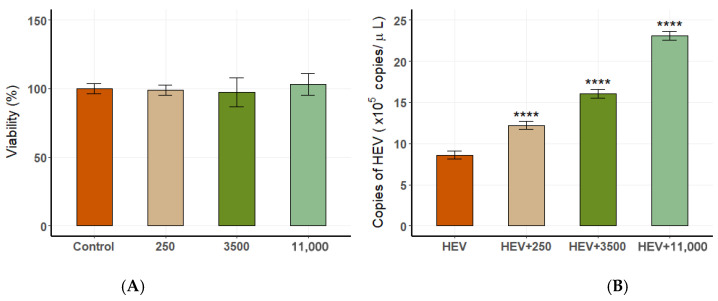
The relationship between estrogen levels and HEV replication in A549 cells. (**A**) Cell viability at different DES concentrations. (**B**) Viral load at varying DES concentrations, showing a positive correlation (**** *p* < 0.0001).

The CCK-8 assay demonstrated that DES concentrations (250 pg/mL, 3500 pg/mL, 11,000 pg/mL) did not affect cell viability (Figure 10A). The results of real-time PCR showed that the initial concentration of the virus solution for the blind transmission of the third generation in A549 cells was 4.88 × 10^2^ copies/μL. After adding different concentrations of DES in the fourth generation, we found that DES concentration was positively correlated with HEV replication. The highest viral load was observed in cells treated with 11,000 pg/mL DES, simulating late pregnancy estrogen levels (Figure 10B). These results indicate that elevated estrogen levels significantly enhance HEV replication during late pregnancy while maintaining cellular viability.

## 4. Discussion

This study successfully established a late-stage pregnant tree shrew model of HEV infection, as well as an in vitro cell infection model, utilizing the zoonotic HEV genotypes 3 and 4 to investigate the influence of estrogen levels on viral replication in both animal and cell-based systems [22]. These models provide novel insights into HEV pathogenesis during pregnancy and contribute to the development of more relevant and accessible HEV infection models in pregnant animals. Furthermore, the findings lay the foundation for elucidating the pathogenic mechanisms of zoonotic HEV genotypes and their interaction with host hormonal regulation. In HEV research, primates are generally considered ideal experimental models due to their close genetic similarity to humans. However, their use is limited by high costs, restricted availability, and ethical concerns. Notably, studies have shown that the intravenous infection of late-pregnant rhesus macaques with HEV does not lead to maternal mortality, preterm delivery, or fetal loss [23,24]. Similarly, although rabbits are susceptible to HEV and exhibit high rates of abortion and maternal death, their greater genetic divergence from humans limits their utility for studying HEV-induced maternal mortality [25]. In contrast, the tree shrew (*Tupaia belangeri*), which is phylogenetically closer to primates, presents several advantages as an experimental model. These include a short reproductive cycle, manageable body size, high encephalization quotient, and low maintenance costs [25]. In this study, the detection of viral load across different tissues revealed that bile contained a particularly high concentration of HEV, consistent with previous reports identifying bile as a high-titer reservoir for the virus [26]. It should be noted, however, that the comparison of viral genome levels across different tissues was based on absolute quantification using standardized input tissue mass (50 mg per sample) and consistent processing volumes. The resulting viral loads were expressed as copies/µL of cDNA template used in qPCR. While this approach supports relative comparison under controlled extraction and amplification conditions, it may not fully reflect tissue-specific viral burden in physiological terms. Factors such as variable RNA yield, tissue density, or innate viral content could affect final measurements. Future studies may benefit from normalization strategies such as expressing viral copies per milligram of tissue or per microgram of total RNA to enhance cross-tissue comparability and data accuracy. Moreover, significant alterations in liver function indicators, such as alanine aminotransferase (ALT) and aspartate aminotransferase (AST)—commonly used biochemical markers of hepatocellular injury—were observed in the infected tree shrews [27]. These changes further validate the suitability of the tree shrew model for studying HEV-induced hepatic damage and its systemic effects during pregnancy. However, one limitation of our model is the lack of specific testing to exclude the presence of endogenous tree shrew hepevirus. Although the HEV strain used was confirmed as genotype 4 of zoonotic origin and all tree shrews were raised in a controlled laboratory environment, the possibility of background infection with tree shrew-specific hepevirus cannot be completely ruled out. Future studies should incorporate molecular assays or sequencing to differentiate between zoonotic HEV and endogenous tree shrew hepevirus to ensure data specificity.

In addition, previous research has shown that organs other than the liver are also affected during HEV infection [28], collectively referred to as extrahepatic manifestations, with cerebral nerve damage being the most common, as well as glomerulonephritis, hematological disorders, pancreatitis, male infertility, and adverse pregnancies in pregnant women [29,30,31]. In the present study, both clinical and histovirological analyses revealed significantly higher rates of abortion and mortality in the HEV-infected group compared to the uninfected controls. Histopathological examination showed classical hepatic lesions consistent with viral hepatitis, along with varying degrees of pathological changes in the uterus and ovaries. These results provide experimental evidence that HEV infection in tree shrews is not restricted to hepatic tissues but may also involve the reproductive organs, potentially via hematogenous spread. Such findings are in agreement with clinical observations in HEV-infected pregnant women, who are at increased risk of miscarriage and preterm labor. This suggests that HEV may negatively impact pregnancy outcomes by impairing placental function or directly damaging the reproductive system. The reproductive pathology observed in our experimental model further supports the hypothesis that HEV possesses a broader tissue tropism and may contribute to adverse gestational outcomes through extrahepatic mechanisms.

The high mortality associated with HEV infection in pregnant women has been linked to alterations in sex hormone levels, particularly estrogen [32]. In female animals, estrogen is a key reproductive hormone secreted primarily by the ovaries and placenta. During pregnancy, maternal levels of estrogen, progesterone, and human chorionic gonadotropin (HCG) increase markedly, especially in the third trimester, to support fetal development and maintain pregnancy. Following miscarriage, the expulsion of the placenta leads to a sharp decline in circulating estrogen levels due to the loss of this hormonal source [33]. However, in the present study, we observed an unexpected increase in estrogen levels following miscarriage in HEV-infected animals. Notably, this increase exhibited a positive linear correlation with viral load, suggesting a potential link between elevated estrogen levels and enhanced HEV replication. These findings imply that estrogen may play a facilitative role in HEV pathogenesis. We hypothesize that estrogen may exacerbate the severity of HEV infection by either promoting viral RNA replication or modulating the host’s antiviral immune response, potentially suppressing effective immune clearance. This observation opens a new avenue for exploring the hormone–virus interaction in HEV infection and underscores the need for further studies to elucidate the underlying molecular mechanisms by which estrogen influences HEV replication and disease severity.

Previous studies have reported that estrogen can exert antiviral effects in certain contexts, such as preventing hepatitis C virus (HCV) entry into host cells via matrix metalloproteinase-9 (MMP-9)-mediated cleavage of viral receptors [34]. However, estrogen also possesses complex immunomodulatory properties, and in some contexts, may contribute to immune suppression and even tumorigenesis. In the case of hepatitis E virus (HEV) infection during pregnancy, elevated estrogen levels have been reported to impair antigen-specific cytotoxic T cell responses, thereby delaying viral clearance [35,36]. This estrogen-mediated immunosuppression, coupled with cytokine dysregulation—evidenced by increased levels of TNF-α, IL-6, IFN-γ, and TGF-β1 in pregnant tree shrews—has been associated with adverse pregnancy outcomes [37,38]. In the present study, to further validate the observations from the animal model, we treated A549 cells with exogenous estrogen analogs at concentrations comparable to those seen in late pregnancy. The results revealed a dose-dependent promotion of HEV replication, indicating that elevated estrogen levels can directly enhance viral replication in vitro. This effect is likely mediated through the suppression of innate antiviral immune responses, thereby allowing the virus to evade immune surveillance. Additionally, natural killer (NK) cells, as critical components of the host’s innate immune system, play a pivotal role in the early defense against HEV infection. NK cells exert antiviral effects by producing type I interferons (IFN-α/β) and mediating cytotoxicity against infected cells. Estrogen may impair NK cell function, thereby indirectly facilitating HEV replication. These findings provide a theoretical foundation for further elucidating the mechanisms underlying the high mortality rate of HEV infection during pregnancy. Nevertheless, the precise mechanisms by which HEV leads to mortality following high-level replication remain to be clarified. Potential contributors include direct cytopathic effects of the virus on hepatocytes, immune-mediated liver injury, and an excessive inflammatory response triggered by virus–host interactions. Further investigations are warranted to dissect these mechanisms and identify potential therapeutic targets for HEV-induced pregnancy complications.

In conclusion, this study suggests that pregnancy-associated hormonal fluctuations and immune modulation may influence the pathogenesis of HEV infection, highlighting the need for further comparative studies involving non-pregnant HEV-infected tree shrews. This study underscores the critical role of estrogen-related hormonal fluctuations and immune modulation in the pathogenesis of HEV during pregnancy. These findings offer valuable insights into the complex interplay between hormonal regulation, host immune responses, and viral replication. A deeper understanding of these mechanisms may contribute to the development of effective strategies for the prevention and control of HEV infection, ultimately aiming to reduce adverse pregnancy outcomes and improve maternal health.

## Data Availability

Data are available in the manuscript.

## Abbreviations

The following abbreviations are used in this manuscript:

HEV	hepatitis E virus
AST	Aspartate aminotransferase
ALT	Alanine aminotransferase
A549	human lung cancer cells
Huh-7	human hepatocellular carcinoma cells
DES	Diethylstilbestrol tablets

## References

[1] Aggarwal R., Krawczynski K. (2000). Hepatitis E: An overview and recent advances in clinical and laboratory research. J. Gastroenterol. Hepatol..

[2] Berglov A., Hallager S., Weis N. (2019). Hepatitis E during pregnancy: Maternal and foetal case-fatality rates and adverse outcomes-a systematic review. J. Viral Hepat..

[3] Khuroo M.S., Kamili S. (2003). Aetiology, clinical course and outcome of sporadic acute viral hepatitis in pregnancy. J. Viral Hepat..

[4] Rein D.B., Stevens G.A., Theaker J., Wittenborn J.S., Wiersma S.T. (2012). The global burden of hepatitis E virus genotypes 1 and 2 in 2005. Hepatology.

[5] Patra S., Kumar A., Trivedi S.S., Puri M., Sarin S.K. (2007). Maternal and fetal outcomes in pregnant women with acute hepatitis E virus infection. Ann. Intern. Med..

[6] Krain L.J., Nelson K.E., Labrique A.B. (2014). Host immune status and response to hepatitis E virus infection. Clin. Microbiol. Rev..

[7] Krawczynski K., Aggarwal R., Kamili S. (2000). Hepatitis E. Infect. Dis. Clin. North. Am..

[8] Aslan A.T., Balaban H.Y. (2020). Hepatitis E virus: Epidemiology, diagnosis, clinical manifestations, and treatment. World J. Gastroenterol..

[9] Lhomme S., Marion O., Abravanel F., Chapuy-Regaud S., Kamar N., Izopet J. (2016). Hepatitis E Pathogenesis. Viruses.

[10] Wu C., Wu X., Xia J. (2020). Hepatitis E virus infection during pregnancy. Virol. J..

[11] Bhatnagar N., Prakash S., Ramakrishna V., Khan D.N., Shrivastava S.S., Venkatesh V., Reddy D.H., Jain A. (2022). Molecular characterisation of hepatitis E virus isolates from north India. Indian. J. Med. Microbiol..

[12] Navaneethan U., Al Mohajer M., Shata M.T. (2008). Hepatitis E and pregnancy: Understanding the pathogenesis. Liver Int. Off. J. Int. Assoc. Study Liver.

[13] Bose P.D., Das B.C., Kumar A., Gondal R., Kumar D., Kar P. (2011). High viral load and deregulation of the progesterone receptor signaling pathway: Association with hepatitis E-related poor pregnancy outcome. J. Hepatol..

[14] Pal R., Aggarwal R., Naik S.R., Das V., Das S., Naik S. (2005). Immunological alterations in pregnant women with acute hepatitis E. J. Gastroenterol. Hepatol..

[15] Bi Y., Yang C., Yu W., Zhao X., Zhao C., He Z., Jing S., Wang H., Huang F. (2015). Pregnancy serum facilitates hepatitis E virus replication in vitro. J. Gen. Virol..

[16] Singh S., Daga M.K., Kumar A., Husain S.A., Kar P. (2019). Role of oestrogen and its receptors in HEV-associated feto-maternal outcomes. Liver Int. Off. J. Int. Assoc. Study Liver.

[17] Tamada Y., Yano K., Yatsuhashi H., Inoue O., Mawatari F., Ishibashi H. (2004). Consumption of wild boar linked to cases of hepatitis E. J. Hepatol..

[18] Pallerla S.R., Harms D., Johne R., Todt D., Steinmann E., Schemmerer M., Wenzel J.J., Hofmann J., Shih J.W.K., Wedemeyer H. (2020). Hepatitis E Virus Infection: Circulation, Molecular Epidemiology, and Impact on Global Health. Pathogens.

[19] Haipeng L., Yanan D. (2023). Relationship between thyroid hormone, estrogen, progesterone and pregnancy in pregnant women. Health Prot. Promot..

[20] Jothikumar N., Cromeans T.L., Robertson B.H., Meng X.J., Hill V.R. (2006). A broadly reactive one-step real-time RT-PCR assay for rapid and sensitive detection of hepatitis E virus. J. Virol. Methods.

[21] Huang F.F., Haqshenas G., Guenette D.K., Halbur P.G., Schommer S.K., Pierson F.W., Toth T.E., Meng X.J. (2002). Detection by reverse transcription-PCR and genetic characterization of field isolates of swine hepatitis E virus from pigs in different geographic regions of the United States. J. Clin. Microbiol..

[22] Smith D.B., Izopet J., Nicot F., Simmonds P., Jameel S., Meng X.J., Norder H., Okamoto H., van der Poel W.H.M., Reuter G. (2020). Update: Proposed reference sequences for subtypes of hepatitis E virus (species Orthohepevirus A). J. Gen. Virol..

[23] Lhomme S., Legrand-Abravanel F., Kamar N., Izopet J. (2019). Screening, diagnosis and risks associated with Hepatitis E virus infection. Expert Rev. Anti Infect. Ther..

[24] Yang F.M., Duan S.Q., Guo Y.Q., Li Y.Y., Yoshizaki S., Takeda N., Wakita T., Muramatsu M., Zhao Y., He Z.L. (2019). Current status of hepatitis E virus infection at a rhesus monkey farm in China. Vet. Microbiol..

[25] Xia J., Liu L., Wang L., Zhang Y., Zeng H., Liu P., Zou Q., Wang L., Zhuang H. (2015). Experimental infection of pregnant rabbits with hepatitis E virus demonstrating high mortality and vertical transmission. J. Viral Hepat..

[26] Wang L., Liang C.N., Li X.B., Wang J., Fu R., Xing J., Shu J.Y., Zhao C.Y., Huang W.J. (2021). Prevalence of Hepatitis E Virus Infection among Laboratory Rabbits in China. Pathogens.

[27] Wang Y., Liu H., Liu S., Yang C., Jiang Y., Wang S., Liu A., Peppelenbosch M.P., Kamar N., Pan Q. (2019). Incidence, predictors and prognosis of genotype 4 hepatitis E related liver failure: A tertiary nested case-control study. Liver Int. Off. J. Int. Assoc. Study Liver.

[28] Pérez-Gracia M.T., Suay-García B., Mateos-Lindemann M.L. (2017). Hepatitis E and pregnancy: Current state. Rev. Med. Virol..

[29] Huang F., Long F., Yu W., Situ J., Fu L., He Z., Dong H., Yang C., Li Y., Yang F. (2018). High prevalence of hepatitis E virus in semen of infertile male and causes testis damage. Gut.

[30] Kamar N., Mansuy J.M., Esposito L., Legrand-Abravanel F., Peron J.M., Durand D., Rostaing L., Izopet J. (2005). Acute hepatitis and renal function impairment related to infection by hepatitis E virus in a renal allograft recipient. Am. J. Kidney Dis..

[31] Li C., Wang H.F. (2011). Hepatitis E virus-related acute liver failure associated with pure red cell aplasia. Hepatobiliary Pancreat. Dis. Int..

[32] Liang J., Shang Y. (2013). Estrogen and cancer. Annu. Rev. Physiol..

[33] Pérez-López F.R., Chedraui P., Troyano-Luque J.M. (2010). Peri- and post-menopausal incidental adnexal masses and the risk of sporadic ovarian malignancy: New insights and clinical management. Gynecol. Endocrinol. Off. J. Int. Soc. Gynecol. Endocrinol..

[34] Ulitzky L., Lafer M.M., KuKuruga M.A., Silberstein E., Cehan N., Taylor D.R. (2016). A new signaling pathway for HCV inhibition by estrogen: GPR30 activation leads to cleavage of occludin by MMP-9. PLoS ONE.

[35] Wasmuth H.E., Lammert F., Matern S. (2003). Genetic risk factors for hepatic fibrosis in chronic liver diseases. Med. Klin..

[36] Gong S.L., Hao X.H., Bi Y.H., Yang C.C., Wang W.J., Mickael H.K., Zhang Y.K., Chen S.F., Qian Z.Y., Huang F. (2021). Hepatitis E viral infection regulates estrogen signaling pathways: Inhibition of the cAMPK-PKA-CREB and PI3K-AKT-mTOR signaling pathways. J. Med. Virol..

[37] Kumar A., Devi S.G., Kar P., Agarwal S., Husain S.A., Gupta R.K., Sharma S. (2014). Association of cytokines in hepatitis E with pregnancy outcome. Cytokine.

[38] Jilani N., Das B.C., Husain S.A., Baweja U.K., Chattopadhya D., Gupta R.K., Sardana S., Kar P. (2007). Hepatitis E virus infection and fulminant hepatic failure during pregnancy. J. Gastroenterol. Hepatol..

